# Use of *Mycobacterium smegmatis* Deficient in ADP-Ribosyltransferase as Surrogate for *Mycobacterium tuberculosis* in Drug Testing and Mutation Analysis

**DOI:** 10.1371/journal.pone.0122076

**Published:** 2015-04-13

**Authors:** Priyanka Agrawal, Sandeep Miryala, Umesh Varshney

**Affiliations:** 1 Department of Microbiology and Cell Biology, Indian Institute of Science, Bangalore, 560012, India; 2 Jawaharlal Nehru Centre for Advanced Scientific Research, Bangalore, 560064, India; University of Padova, Medical School, ITALY

## Abstract

Rifampicin (Rif) is a first line drug used for tuberculosis treatment. However, the emergence of drug resistant strains has necessitated synthesis and testing of newer analogs of Rif. *Mycobacterium smegmatis* is often used as a surrogate for *M*. *tuberculosis*. However, the presence of an ADP ribosyltransferase (Arr) in *M*. *smegmatis* inactivates Rif, rendering it impractical for screening of Rif analogs or other compounds when used in conjunction with them (Rif/Rif analogs). Rifampicin is also used in studying the role of various DNA repair enzymes by analyzing mutations in RpoB (a subunit of RNA polymerase) causing Rif resistance. These analyses use high concentrations of Rif when *M*. *smegmatis* is used as model. Here, we have generated *M*. *smegmatis* strains by deleting *arr* (*Δarr*). The *M*. *smegmatis Δarr* strains show minimum inhibitory concentration (MIC) for Rif which is similar to that for *M*. *tuberculosis*. The MICs for isoniazid, pyrazinamide, ethambutol, ciprofloxacin and streptomycin were essentially unaltered for *M*. *smegmatis Δarr*. The growth profiles and mutation spectrum of *Δarr* and, *Δarr* combined with *ΔudgB* (*udgB* encodes a DNA repair enzyme that excises uracil) strains were similar to their counterparts wild-type for *arr*. However, the mutation spectrum of *ΔfpgΔarr* strain differed somewhat from that of the *Δfpg* strain (*fpg* encodes a DNA repair enzyme that excises 8-oxo-G). Our studies suggest *M*. *smegmatis Δarr* strain as an ideal model system in drug testing and mutation spectrum determination in DNA repair studies.

## Introduction

*Mycobacterium tuberculosis* causes tuberculosis (TB) in people of all ages worldwide. Multi-drug therapy involving rifampicin (Rif), isoniazid, pyrizinamide, ethambutol and streptomycin is used for TB treatment [1]. However, conditions such as noncompliance of the patients to the drug regimen during the protracted periods (six to nine months) of treatment, consequences of the host/pathogen interaction and the niches in which the pathogen survives have resulted in the emergence of multi drug resistance (MDR), extensively drug resistance (XDR) and totally drug resistance (TDR) cases of TB [2–5]. Thus, a search for newer drugs is an active area of TB research. Rif is the most effective drug against TB [6, 7]. The need for effective drug therapy has led to synthesis of many of its structural analogs *e*. *g*. rifabutin, rifapentine, rifametane (SPA-S-565), rifalazil, CPG 7040 [8]. To increase the repertoire of Rif analogs, reverse genetics approches of selective engineering of the rifamycin polyketide synthase gene cluster modules with those from the rapamycin polyketide synthase gene cluster have also been used successfully [6, 7]. Initial screenings of such compounds require a simple and rapid bioassay to avoid queuing for biological testing.

*M*. *smegmatis* serves as a good model system to understand the physiology of *M*. *tuberculosis* [9]. However, checking efficacies of Rif analogs, or other compounds/derivatives in combination with Rif is not possible in *M*. *smegmatis* as it possesses an ADP-ribosyltransferase (Arr, encoded by *arr* gene), which ADP-ribosylates Rif and renders it ineffective [10]. Arr recognizes the hydroxyl group on the ansamycin bridge of Rif and transfers ADP ribose group to this oxygen using NAD as a co-substrate [11]. Such a bioconversion of Rif leads to higher minimum inhibitory concentrations (MICs) in *M*. *smegmatis* compared to those reported for *M*. *tuberculosis*. Another mechanism that causes a low level increase in Rif resistance in mycobacteria is via an RNA polymerase binding protein, termed RbpA [12]. This protein, however, occurs in common in both *M*. *tuberculosis* (Rv2050) and *M*. *smegmatis* (MSMEG_3858) and is required for their optimal growth [12, 13]. Thus, it is desirable to generate a strain of *M*. *smegmatis*, which may offer MICs for Rif or its derivatives in a range similar to those reported for *M*. *tuberculosis*. The Rif and its analogs will not be ADP ribosylated in *M*. *smegmatis* if it lacked the *arr* gene.

The primary mode of action of Rif is by targeting β subunit of RNA polymerase (*rpoB*). Rif resistance in *M*. *tuberculosis* or in *M*. *smegmatis* (at higher doses) arises due to mutations in *rpoB* [14, 15]. The mutations in *rpoB* leading to Rif resistance are predominantly localised to a highly conserved region of 27 amino acids (81 nucleotides) called RRDR (**R**ifampicin **R**esistance **D**etermining **R**egion). This observation has been exploited to isolate and characterize mutations in the RRDR to understand the mechanism of mutation occurrence due to deficient DNA repair and replication. A deficiency of DNA repair function results in increased occurrence of mutations in general, which in turn facilitates isolation of Rif resistant mutants. Sequence analysis of the RRDR in the Rif resistant mutants allows for identification of specific base changes, and in better understanding of the mechanism of a DNA repair enzymes in avoidance of specific mutations [16–18].

In this study, we have generated a *M*. *smegmatis* strain wherein *arr* gene was knocked out. The strain was susceptible to Rif with MIC as low as that reported for *M*. *tuberculosis*. Furthermore, we tested the utility of this strain in mutation spectrum determination arising as a result of deficiency of DNA repair enzymes such as UdgB, and Fpg. UdgB and Fpg are involved in base excision repair of uracil and 8-oxo-guanine arising from deamination of cytosine, and oxidation of guanine, respectively.

## Materials and Methods

### Strains, plasmids, and DNA oligomers

The bacterial strains/plasmids and the DNA oligomers used in this study are described in Tables 1 and 2, respectively.

**Table 1:** The list of strains and plasmids used in the study.

**Strains/plasmids**	**Description of the strains**	**References**
*mc* ^*2*^ *155*	High efficiency transformation strain of *M*. *smegmatis*.	[19]
*Δarr*::*kan*	*M*. *smegmatis mc* ^*2*^ *155* where *arr* gene is disrupted with *kan* ^R^ cassette	This study
*ΔudgB*::*hyg*	*M*. *smegmatis mc* ^*2*^ *155* where *udgB* gene is disrupted with *hyg* ^R^ cassette	[20]
*Δfpg*::*hyg*	*M*. *smegmatis mc* ^*2*^ *155* where *fpg* gene is disrupted with *hyg* ^R^ cassette	[21]
*ΔudgB*::*hygΔarr*::*kan*	*M*. *smegmatis mc* ^*2*^ *155* where *udgB* and *arr* genes are disrupted with *hyg* ^R^ and *kan* ^R^ cassettes, respectively	This study
*Δfpg*::*hygΔarr*::*kan*	*M*. *smegmatis mc* ^*2*^ *155* where *fpg* and *arr* genes are disrupted using *hyg* ^R^ and *kan* ^R^ cassettes, respectively	This study
*E*. *coli TG1*	*E*. *coli* K-12 *supE thi-1Δ (lac-proAB) Δ (mcr-hsdSM)*	[22]
pJET 1.2	PCR product cloning vector for *E*. *coli* (Amp^R^)	Thermo Scientific
pPR27 (Gen^R^)	A mycobacterial-*E*. *coli* shuttle vector with *sacB* selection marker, mycobacterial origin of replication being temperature sensitive and gentamycin resistance cassette	[23]
pUC4K	An *E*. *coli* multicopy plasmid with kanamycin resistance cassette	[24]
pMV361(Hyg^R^)	A vector with origin for replication in *E*. *coli* and an attachment site *attP* and integrase gene *int* for integration into L5 site in mycobacteria, and *hsp60* promoter.	[25]
pMV261(Hyg^R^)	A multi-copy vector system with origins for replication in *E*. *coli* and mycobacteria (pAL500), and *hsp60* promoter.	[25]

**Table 2:** DNA oligomer used in the study.

**Name of the oligomer**	**Sequence of the oligo (5’ to 3’)**
arr_up_fp	GACCATCTAGACCAGACCGGC
arr_down_rp	AACCATCTAGACCGCCCTCGA
arr_HindIII_rp	GAAGCTTCTAGTCATAGATGACCG
arr_ sc_comp_PvuII_fp	GCCGGCAGCTGCGCGAACGCGTCGATGCC
Kan_saci fp	GATGAGAGCTCTGTTGTAGGTGGAC
Kan_saci rp	TGAGAGAGCTCACTCATTA GGCACC
306-rpoB-fp	CGACCACTTCGGCAACCG
306-rpoB-rp	CGATCAGACCGATGTTGG
Msm-*rpoB*-seq-Fp	GTCTGCGCACCGTCGGTG

### Growth conditions

The media components were purchased from Difco. Other chemicals were from Sigma except hygromycin which was purchased from Calbiochem. Enzymes used for DNA manipulations were from Thermo Fischer and New England Biolabs. *E*. *coli* was grown in LB media supplemented with different antibiotics when required. The concentrations of antibiotics used for *E*. *coli* were ampicillin (Amp), 100 μg/ml; kanamycin (Kan), 25 μg/ml; and gentamycin (Gm), 20 μg/ml. *M*. *smegmatis* was grown either in LB supplemented with 0.2% Tween-80 (v/v) (LBT) or in 7H9 media with 0.2% glycerol (v/v) and 0.2% Tween-80 (v/v). Agar (1.6%, w/v) and Tween-80 (0.05%, v/v) were added for solid media. The concentrations of different antibiotics used for *M*. *smegmatis* were Kan, 50 μg/ml; Gm, 5 μg/ml; and hygromycin (Hyg), 50 μg/ml. Rifampicin (Rif) was used at variable concentrations as mentioned. Unless mentioned otherwise, strains were grown at 37°C.

### Generation of *arr* knockout construct and the strain

DNA oligomers for PCR were designed 964 bp upstream (arr_up_fp) and 973 bp downstream (arr_down_rp) of the *arr* open reading frame (ORF). To amplify *arr*, ~100 ng *M*. *smegmatis* mc^2^ 155 genomic DNA and 1 pmol each of arr_up_fp and arr_down_rp were used with *Pfu* DNA polymerase (1U) in 1X *Pfu* buffer (200 mM Tris-HCl, pH 8.8 at 25°C), 100 mM (NH_4_)_2_SO_4_, 100 mM KCl, 1 mg/mL BSA, 1% (v/v) Triton X-100, 20 mM MgSO_4_) and 1 mM dNTPs in a 20 μl reaction. The reaction conditions included initial denaturation at 94°C for 5 min, followed by 30 cycles of heating at 94°C for 1 min, 68°C for 30 s, and 70°C for 3 min. The reaction was then heated at 70°C for 10 min. The 2.3 kb amplicon thus obtained was ligated to pJET 1.2 to generate pJET_arr.

The kanamycin resistance cassette (*kan* ^R^) was PCR amplified from pUC4k (~40 ng) using 1 pmol each of Kan_saci fp and Kan_saci rp with *Pfu* DNA polymerase as above in 20 μl reaction. The reaction condition included initial denaturation at 94°C for 4 min, followed by 30 cycles of heating at 94°C for 1 min, annealing at 55°C for 45 s, extension at 70°C for 3 min followed by a final extension at 70°C for 10 min. The PCR product (1.27 kb) was gel eluted and digested with SacI, and cloned into similarly digested pJET_arr to generate pJET_arr_ko_kan. As three SacI sites are present in the ORF of *arr* (MSMEG_1221), this procedure resulted in deletion of a major part of the ORF (deletion of 330 bp out of the 429 bp long ORF). The first 66 and the last 33 nucleotides were still retained to ensure that *arr* deletion did not impact the neighboring genes. The pJET_arr_ko_kan was digested with XbaI to release a 3.2 kb *arr* allele exchange substrate (wherein the *kan* ^R^ marker is flanked by the upstream and downstream regions of *arr* and subcloned into similarly digested *E*. *coli*-mycobacteria shuttle vector, pPR27. The final plasmid, pPR*Δarr*::*kan* was then electroporated into *M*. *smegmatis* mc^2^155, and its two derivatives namely, *ΔudgB*::*hyg* and *Δfpg*::*hyg*. The transformants were selected on Gm and Kan plates and grown at 30°C. The colonies so obtained were inoculated in LBT containing Kan and grown at 30°C to saturation. Culture (1 ml) was then plated on Kan plate with 10% sucrose and incubated at 39°C. The colonies that appeared were patched on 10% sucrose plates containing Kan and Gm, respectively. The colonies that grew only on Kan plate but not on Gm plate were screened further by PCR using *arr* flanking primers arr_up_fp and arr_down_rp.

### Generation of *arr* expression plasmids

The *arr* gene was PCR amplified in a reaction (20 μl) containing ~100 ng *M*. *smegmatis* genomic DNA, 10 pmol each of arr_up_fp and arr_HindIII_rp and *Pfu* DNA polymerase as above for generation of the knockout constructs. The reaction conditions included initial heating at 98°C for 4 min, followed by 30 cycles of heating at 98°C for 1 min, 64°C for 40 s, 70°C for 3 min followed by a final extension at 70°C for 10 min. The amplicon of 1.4 kb so obtained was digested with MscI and HindIII, and cloned into similarly digested pMV261 plasmid to generate pMV261*arr*.

For single copy integrative construct, *arr* gene was PCR amplified in a reaction (20 μl) set up as above to contain ~100 ng *M*. *smegmatis* genomic DNA, 10 pmol each of arr_ sc_comp_PvuII_fp and arr_HindIII_rp and *Pfu* DNA polymerase. The reaction was heated at 98°C for 4 min followed by 30 cycles of heating at 98°C for 1 min, 64°C for 45 s, 70°C for 3 min followed by a final extension at 70°C for 10 min. The amplicon of 883 bp was digested with PvuII and HindIII, and cloned into similarly digested pMV361 to generate pMV361*arr*.

### MIC determinations

Wells of a 96 well microtitre plate were filled with 50 μl of 7H9 media (in case of pyrazinamide pH was adjusted to 5.5) except for in the first column. Double the required antibiotic concentration was prepared and 100 μl volumes were added to the first column. This was serially diluted to half the concentration by mixing with media only in the subsequent wells till the last second column. Last column was a no antibiotic control. The *M*. *smegmatis* strains were grown in replicates in 7H9 medium to an OD_600nm_ of 0.6, diluted 1000 times, and 50 μl of the diluted culture was added to each well such that the final concentration of antibiotic in the first well comes down to the desired concentration. The plate was sealed with parafilm to avoid drying of the cultures, and incubated under mild shaking (100 RPM) conditions in *Innova 4230* incubator shaker at 37°C for 40 h. After 40 h of incubation, 30 μl of resazurin dye (filter sterilized, 0.2 mg/ml concentration) was added to each well and incubated for 6 h under shaking and imaged. MICs were determined as the values of the first well showing no growth as indicated by resazurin dye staining.

### Effects of acidified nitrite and oxidative stress on growth of the bacteria

Isolated colonies of different strains were inoculated in triplicate in 7H9 media containing 0.5% Tween-80. The cultures were grown to saturation at 37°C for ~50 h and then diluted 100 fold in 7H9 (pH 5.5) supplemented with 0 to 2.0 mM sodium nitrite and seeded in sterile 100 well plates. Growth was monitored at OD_600nm_ every 3 h for 40 h in kinetic growth reader with constant shaking. The data so obtained were plotted using Graph Pad Prism 5 software. To study the effect of oxidative stress, the cultures were grown in duplicate in LBT at 37°C for 48 h (stationary phase culture). An inoculum (1%) from the stationary phase cultures was added to 25 ml LBT and incubated at 37°C under continuous shaking, and either not supplemented or supplemented with 3 mM H_2_O_2_ after 6 h. Culture growth was monitored every 6 h by observing OD_600nm_ of 1 ml culture aliquot for 48 h.

### Mutation spectrum analysis

Aliquots (1 ml) of saturated cultures of *M*. *smegmatis Δarr*::*kan* strains (grown at 37°C for 48 h) were plated on Rif (5 μg/ml) and incubated at 37°C for 3 days. To further increase the efficiency of mutant selection, we introduced a step of secondary selection, wherein the randomly selected colonies that grew on Rif plates were patched on Rif 20 μg/ml. The colonies that grew upon patching were resuspended in 20 μl water, heated to 90°C for 5 min, and centrifuged at 13,000 rpm at 4°C for 5 min. The supernatant was used as source of genomic DNA to amplify RRDR of *rpoB*. To determine the mutation spectrum for *M*. *smegmatis Δfpg* strain, samples were processed as described earlier [21]. The PCR products were sent for sequencing to Macrogen Inc. (S. Korea).

## Results and Discussion

### Generation and phenotypic characterization of the *arr* knockouts

**Fig 1:**
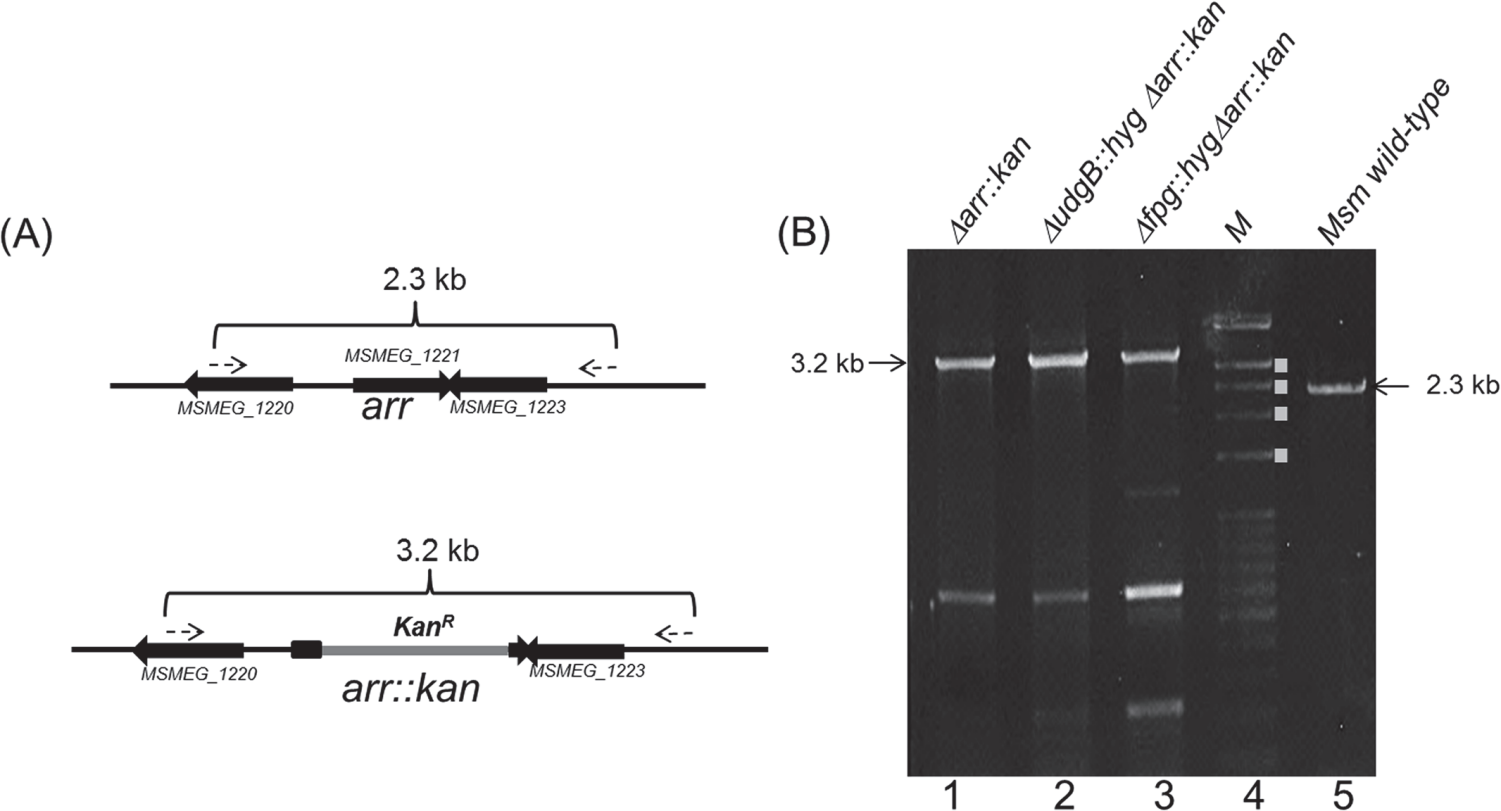
Generation of *MsmΔarr*::*kan* strain. (A) Schematic representation of *arr* gene (MSMEG_1221) locus (not to scale) and its replacement with *kan* ^R^ marker (*arr*::*kan*). As described in the Materials and Methods, the knockout procedure resulted in deletion of 330 bp of the 429 bp of the ORF (from nucleotide position 67 to 396). Primers used for generation and confirmation of the knockout, are represented by dashed arrows. (B) Agarose gel electrophoresis for PCR products for confirmation of amplicon sizes. Lanes: 1, *MsmΔarr*::*kan*; 2, *MsmΔudgB*::*hygΔarr*::*kan*; 3, *MsmΔfpg*::*hygΔarr*::*kan*; 4, DNA size markers (M, bands denoted with white filled rectangles (from top to bottom indicate sizes of ~3.0, 2.5, 2.0 and 1.5 kb); and 5, wild-type *M*. *smegmatis* (*Msm*).

*M*. *smegmatis arr* (MSMEG_1221) knockout construct (pPR*Δarr*::*kan*) was electroporated into various *M*. *smegmatis* strains (wild-type, *ΔudgB*::*hyg* and *Δfpg*::*hyg*) and the putative knockouts were selected as described in Materials and Methods. The knockouts were further characterised by PCR using *arr* flanking primers. The schematic in Fig 1A shows that amplicons of 3.2 kb and 2.3 kb are expected for the *Δarr*::*kan* (also referred to as *Δarr*) and the wild-type alleles, respectively. The results in Fig 1B reveal an expected pattern of the amplicons from the strains wild-type (lane 5) or knockout for *arr* (lanes 1–3).

**Fig 2:**
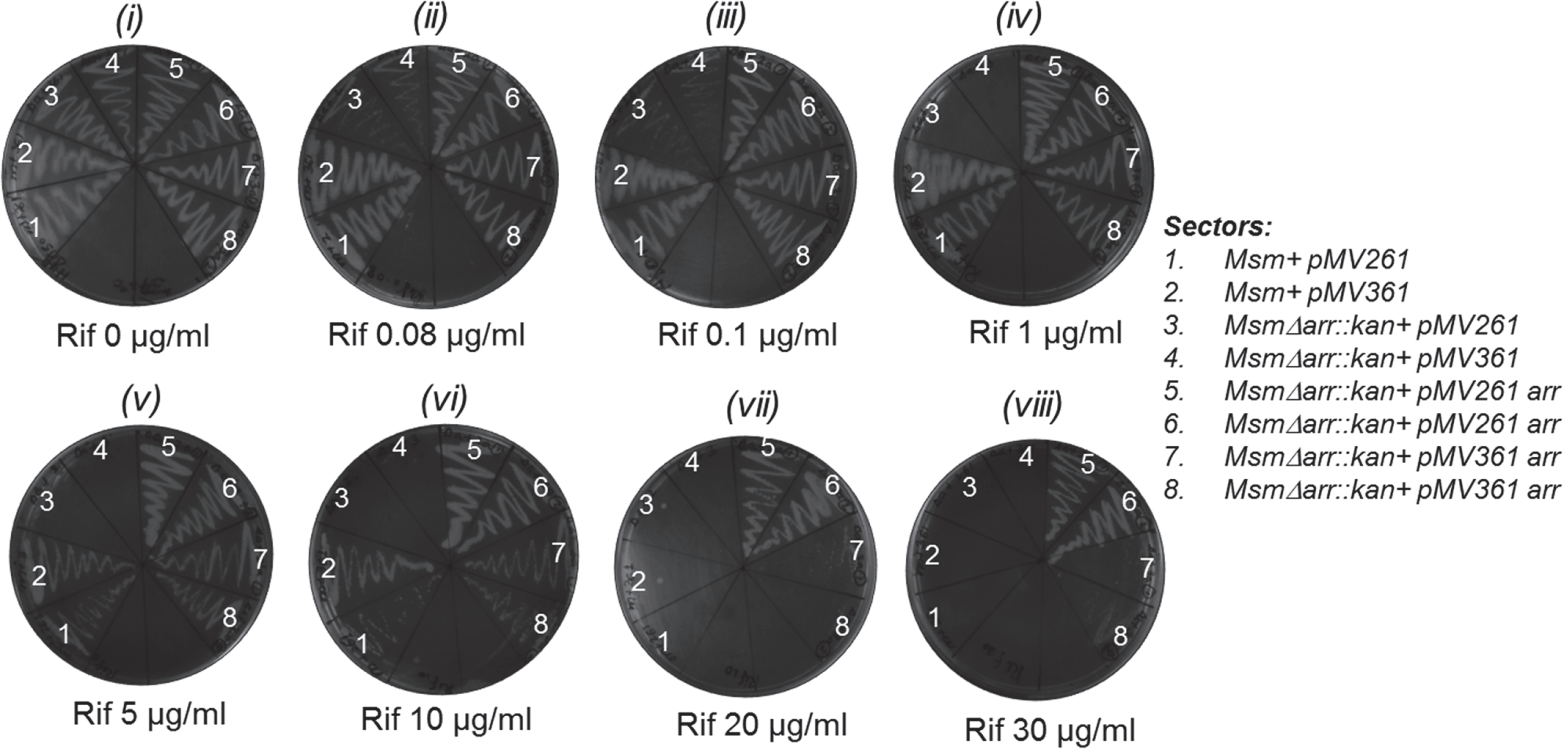
Rifampicin plate assay for confirmation of *arr* knockout and complementation by *arr* gene on an episomal (pMV261) or integrative (pMV361) vector. The bacterial cultures were grown to saturation for 48 h in LBT and streaked on LBT agar plates containing different concentrations of rifampicin as indicated. All plates contained 50 μg/ml hygromycin. Plates were incubated at 37°C for 48 h.

**Fig 3:**
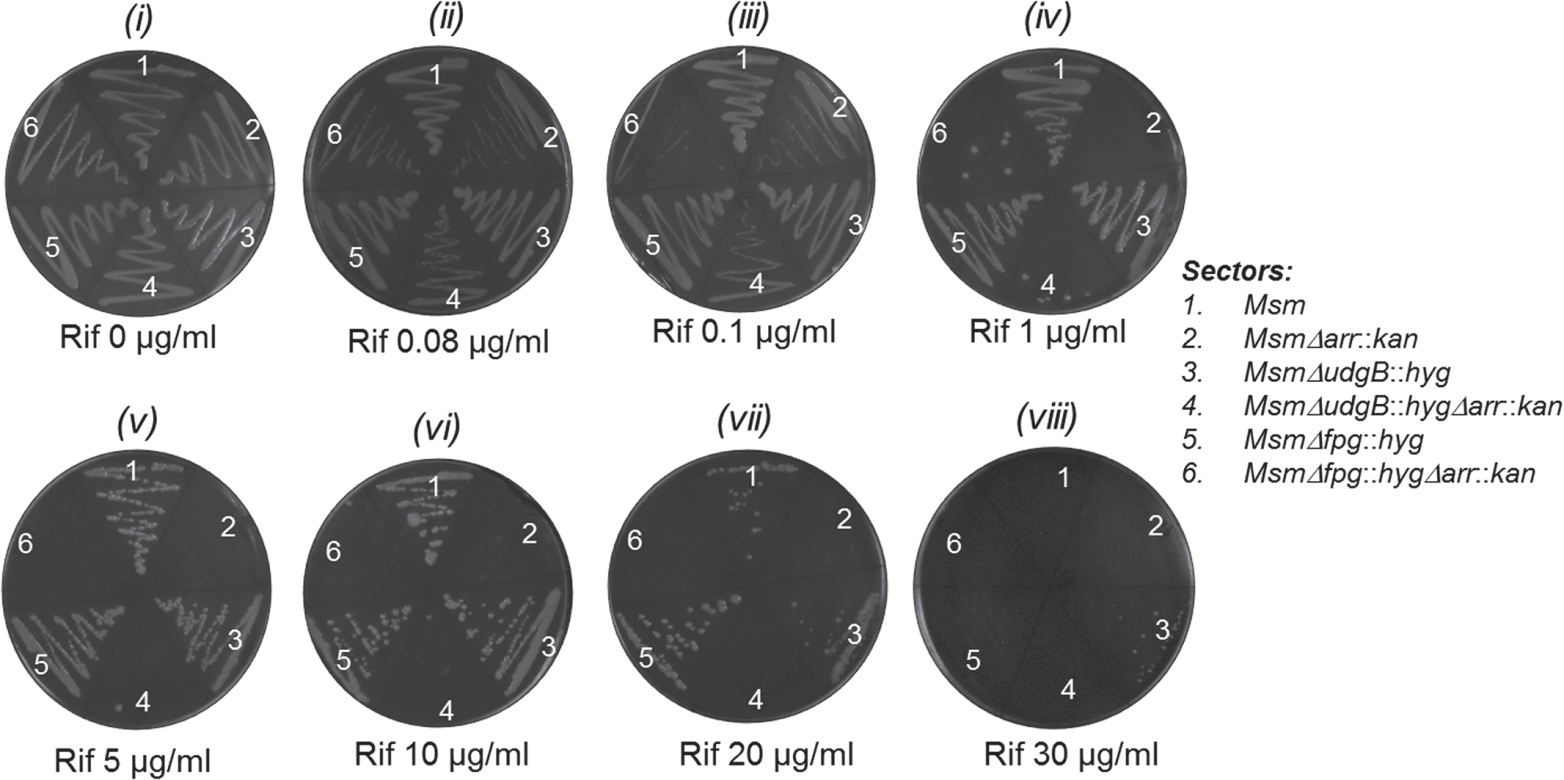
Rifampicin plate assay. Growth of *M*. *smegmatis* wild-type (*Msm*), *ΔudgB*::*hyg*, *Δfpg*::*hyg* and their *Δarr*::*kan* derivatives as detailed in the sectors list.

To check if *arr* deletion rendered *M*. *smegmatis Δarr* strain more susceptible to killing by Rif, we introduced into it either an empty or *arr* containing episomal (pMV261 or pMV261*arr*, Hyg^R^) or integrative (pMV361 or pMV361*arr*, Hyg^R^) plasmid, and streaked the transformants on plates containing Hyg (50 μg/ml) alone or together with varying concentration of Rif. As control, *M*. *smegmatis* strain wild-type for *arr* was also transformed with an empty vector (pMV261 or pMV361). As shown in Fig 2, compared to the wild-type strains (sectors 1 and 2, harboring pMV261 and pMV361, respectively), the knockout (*Δarr*::*kan*) strains (sectors 3 and 4, harboring pMV261 and pMV361, respectively) became susceptible to Rif even at the lowest concentration of 0.08 μg/ml (panel ii). At a concentration of 1 μg/ml (panel iv), no growth was detected even after 48 h of incubation at 37°C. However, when complemented with a single copy *arr* from an integrative construct (sectors 7 and 8), it showed Rif resistance equivalent to the wild-type strain (sectors 1 and 2) of about 10 μg/ml (panel vi). The introduction of multicopy *arr* on an episomal construct (sectors 5 and 6), resulted in elevated resistance of the transformants to Rif concentrations of 20 and 30 μg/ml (compare sector 1 with sectors 5 and 6, panels vii and viii). As control, all strains showed equivalent growth on the plate lacking Rif (panel i). These observations showed that Rif sensitivity of the *Δarr*::*kan* strain is due to the deletion of *arr* and not because of any polar effects due to a change in the genomic locus of *arr*. Rif sensitivity of the *Δarr*::*kan* strain to as low as 0.08 μg/ml is similar to the Rif sensitivity reported for *M*. *tuberculosis*. Consistent with these observations, and as shown in Fig 3, deletion of *arr* in *ΔudgB*::*hyg* and *Δfpg*::*hyg* backgrounds also conferred Rif sensitivities (compare sectors 1, 3, and 5 with sectors 2, 4 and 6, respectively in panels ii to vii). As control, all strains grow equally well on a plate lacking Rif (panel i) and fail to grow at high concentration of 30 μg/ml (panel viii).

### MIC determination of *arr* knockout and the complemented strains towards Rif and other anti-tuberculosis drugs

**Fig 4:**
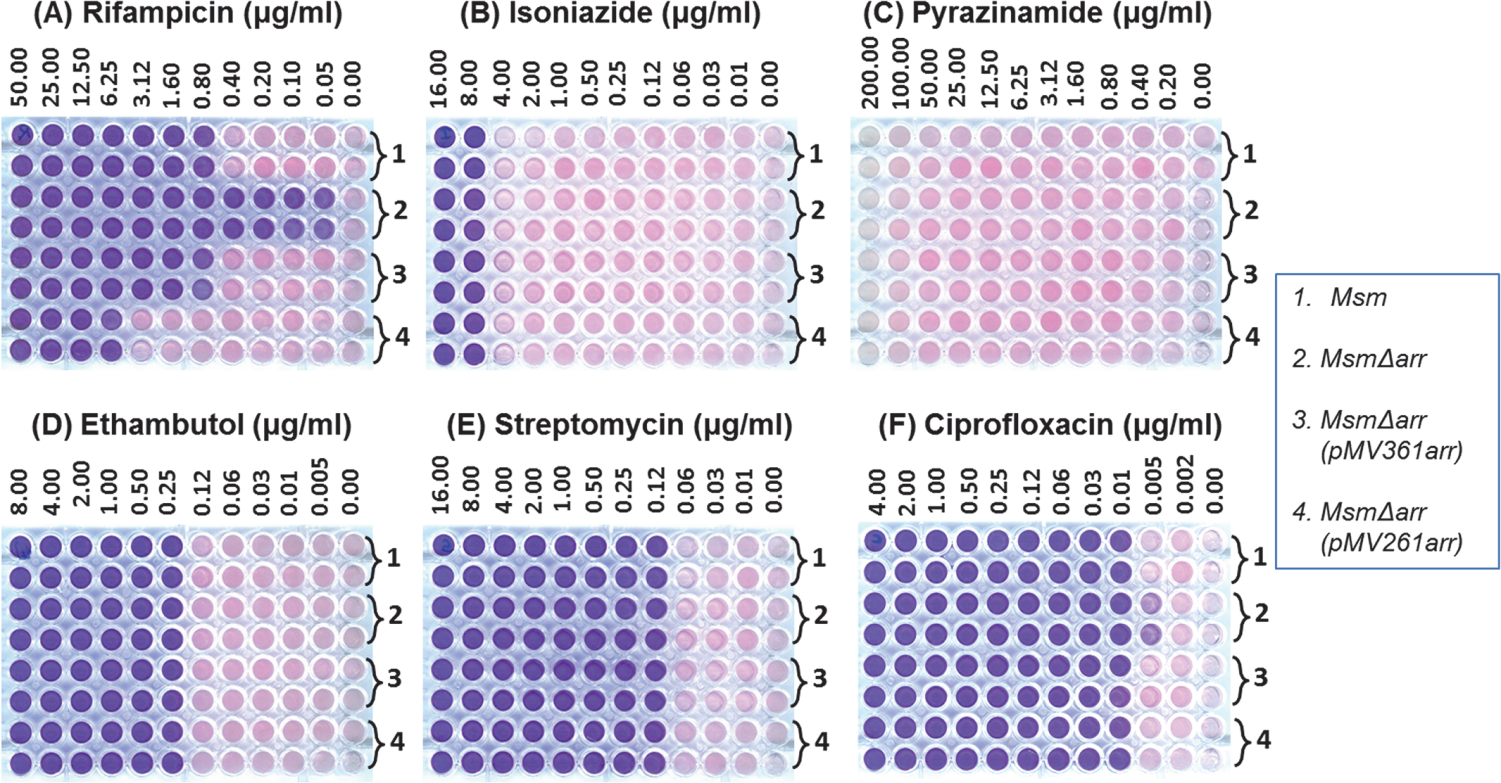
MICs against various drugs as indicated for the different strains. The bacterial cultures were grown till 0.6 OD_600_ and diluted 1000 fold. Aliquots (50 μl) of the diluted culture was added to the serial dilutions of antibiotics (in duplicate rows) and incubated under mild shaking conditions at 37°C for 40 h, after which resazurin was added to each well. The color changes were observed and imaged 6 h after addition of resazurin.

Following our observations in Figs 2 and 3, we determined MICs for TB drugs in a microtitre plate format using resazurin reduction assays (Fig 4). While the MIC of Rif (Fig 4A) for *M*. *smegmatis* was 800 ng/ml that for the *Δarr*::*kan* strain it was <50 ng/ml. The complemented strains followed the same trend observed from the agar plates. The MICs for other antibiotics such as isoniazid, pyrazinamide, ethambutol and streptomycin (Fig 4B and 4E; Table 3), remained unaltered upon deletion of *arr*. On the other hand, we consistently observed a mild increase in the sensitivity of the *Δarr*::*kan* strain to ciprofloxacin (Fig 4F). However, this small difference was invisible once the cultures were incubated for 12 to 18 h following resazurin addition (*data not shown*). A low level increase in the sensitivity of the *Δarr*::*kan* strain to ciprofloxacin may be related to the reported [26] increase in *arr* transcripts in *M*. *smegmatis* treated with the drug. Nonetheless, by and large, the MICs for the other TB drugs for the wild-type and the *Δarr*::*kan* strains were similar. Hence, the *Δarr* strain could serve as an efficient system to check MICs of Rif analogs either alone or in combination with other anti TB drugs.

**Table 3:** MICs for different antibiotics.

Antibiotics Strains	Rifampicin (μg/ml)	Isoniazid (μg/ml)	Pyrazinamide (μg/ml)	Ethambutol (μg/ml)	Streptomycin (μg/ml)	Ciprofloxacin (μg/ml)
***Msm(wt)***	800 ng/ml	8 μg/ml	> 200 μg/ml	250 ng/ml	125 ng/ml	10 ng/ml
***MsmΔarr***	<50 ng/ml	8 μg/ml	> 200 μg/ml	250 ng/ml	125 ng/ml	5 ng/ml
***MsmΔarr+ pMV361 arr***	800 ng/ml	8 μg/ml	> 200 μg/ml	250 ng/ml	125 ng/ml	10 ng/ml
***MsmΔarr+ pMV261 arr***	6.25 μg/ml	8 μg/ml	> 200 μg/ml	250 ng/ml	125 ng/ml	10 ng/ml

### Growth of the *arr* knockout strains in acidified nitrite and oxidative stress

A number of studies in the area of DNA repair involve testing of the effect of reactive nitrogen intermediates or the reactive oxygen species [18]. For these studies, we made use of two of the strains (*Δfpg*::*hyg* and *ΔudgB*::*hyg*) developed earlier in our laboratory as strains defective in base excision repair [20, 21]. UdgB is an enzyme belonging to a superfamily of uracil DNA glycosylases involved in base excision repair of uracils that arise in DNA either due to deamination of cytosines or incorporation of dUMP moieties during DNA replication [20]. On the other hand, Fpg is involved in the base excision repair of 8-oxo-G which arises in DNA due to oxidative stress. Mycobacteria possess G+C rich genomes and are subjected to reactive nitrogen intermediates (RNI) and reactive oxygen species (ROS) in the host. Both RNI and ROS are known to enhance damages of cytosine to uracil and guanine to 8-oxo-guanine.

**Fig 5:**
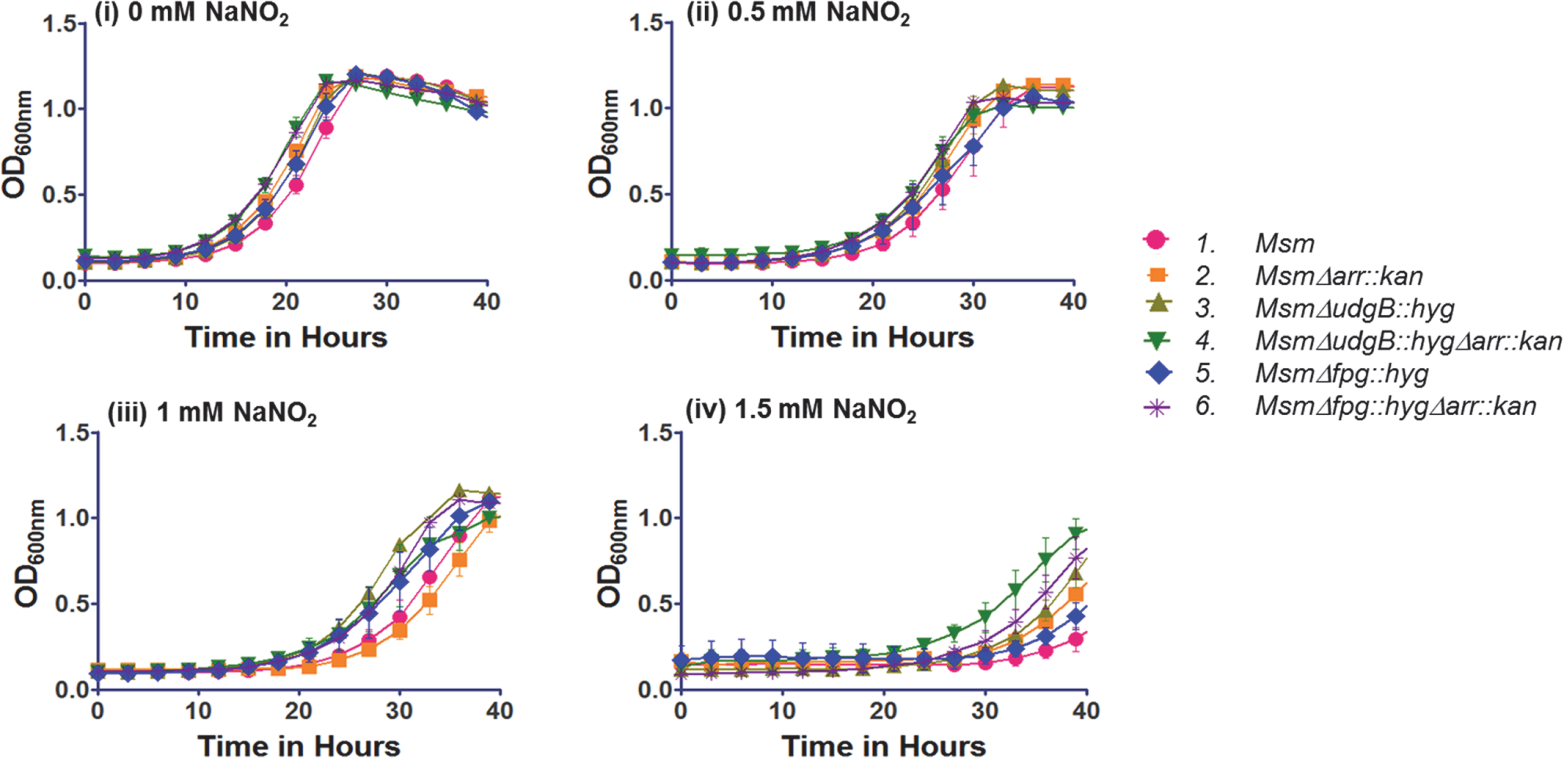
Effect of acidified NaNO_2_ on growth. *M*. *smegmatis* (*Msm*) strains were grown in 7H9 to saturation at 37°C and diluted 100 fold in 7H9 (pH 5.5) either in the absence (0 mM) or presence of (0.5 mM, 1 mM, or 1.5 mM) NaNO_2_. The growth was monitored as OD at 600 nm in Bioscreen kinetic growth reader at intervals of 3 h for 40 h. The growth was plotted using GraphPad prism 5 software.

**Fig 6:**
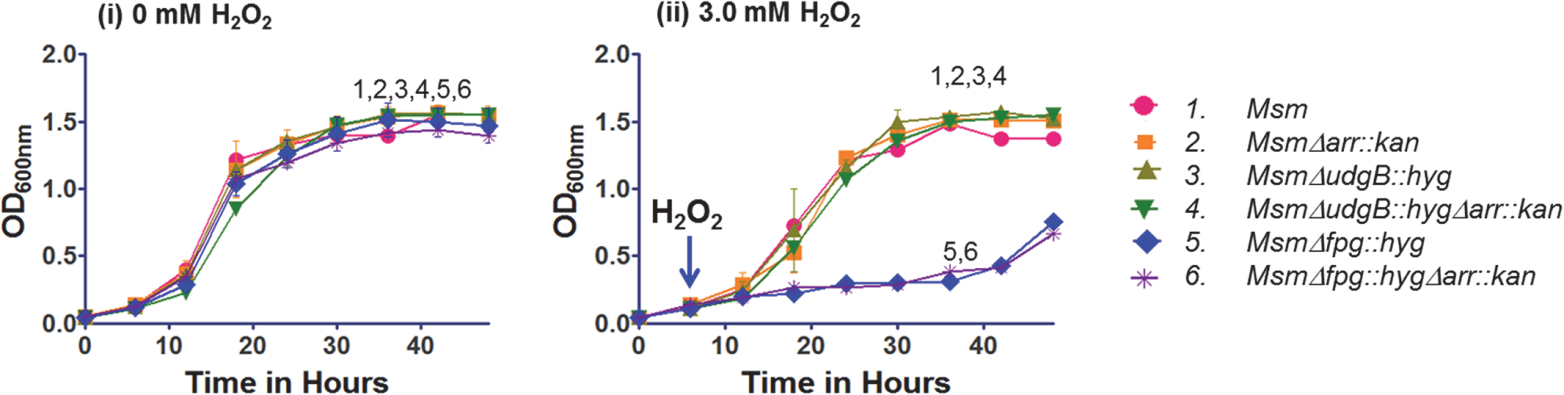
Effect of H_2_O_2_ on growth. *M*. *smegmatis* (*Msm*) strains were grown to saturation in LBT at 37°C and subcultured with 1% inoculum in 25 ml LBT, grown for 6 h and grown further either in the absence (0 mM) or presence (3 mM) H_2_O_2_. The growth was monitored by taking OD readings at 600 nm, at time intervals of every 6 h for 48 h.

Thus, we investigated the effect of acidified sodium nitrite (to mimic RNI) and hydrogen peroxide (to mimic ROS) on the growth of the *Δarr* strains. As shown in Fig 5 (panels i-iv), the *Δarr* strains grew similar to their counterparts wild-type for *arr* when subjected to the acidified sodium nitrite. Further, when we subjected the strains to the oxidative stress by inclusion of hydrogen peroxide in the medium (Fig 6), as expected, compared to the wild-type strain (*M*. *smegmatis* mc^2^155), the *Δfpg*::*hyg* strain showed increased susceptibility to hydrogen peroxide [21]. More importantly, the *Δarr*::*kan* strains grew similar to their counterparts wild-type for *arr*. For example, compare *M*. *smegmatis* mc^2^155 with *M*. *smegmatis Δarr*::*kan*, *M*. *smegmatis ΔudgB*::*hyg* to *M*. *smegmatis ΔudgB*::*hygΔarr*::*kan* and *M*. *smegmatis Δfpg*::*hyg* to *M*. *smegmatis Δfpg*::*hygΔarr*::*kan*. These observations are consistent with the earlier report that the expression of DNA repair functions in *Δarr* strain (with respect to its wild-type counterpart) did not reveal significant changes, despite the fact that in the wild-type strain, Arr expression was upregulated under the stress conditions [26].

### Mutation spectrum analysis

**Fig 7:**
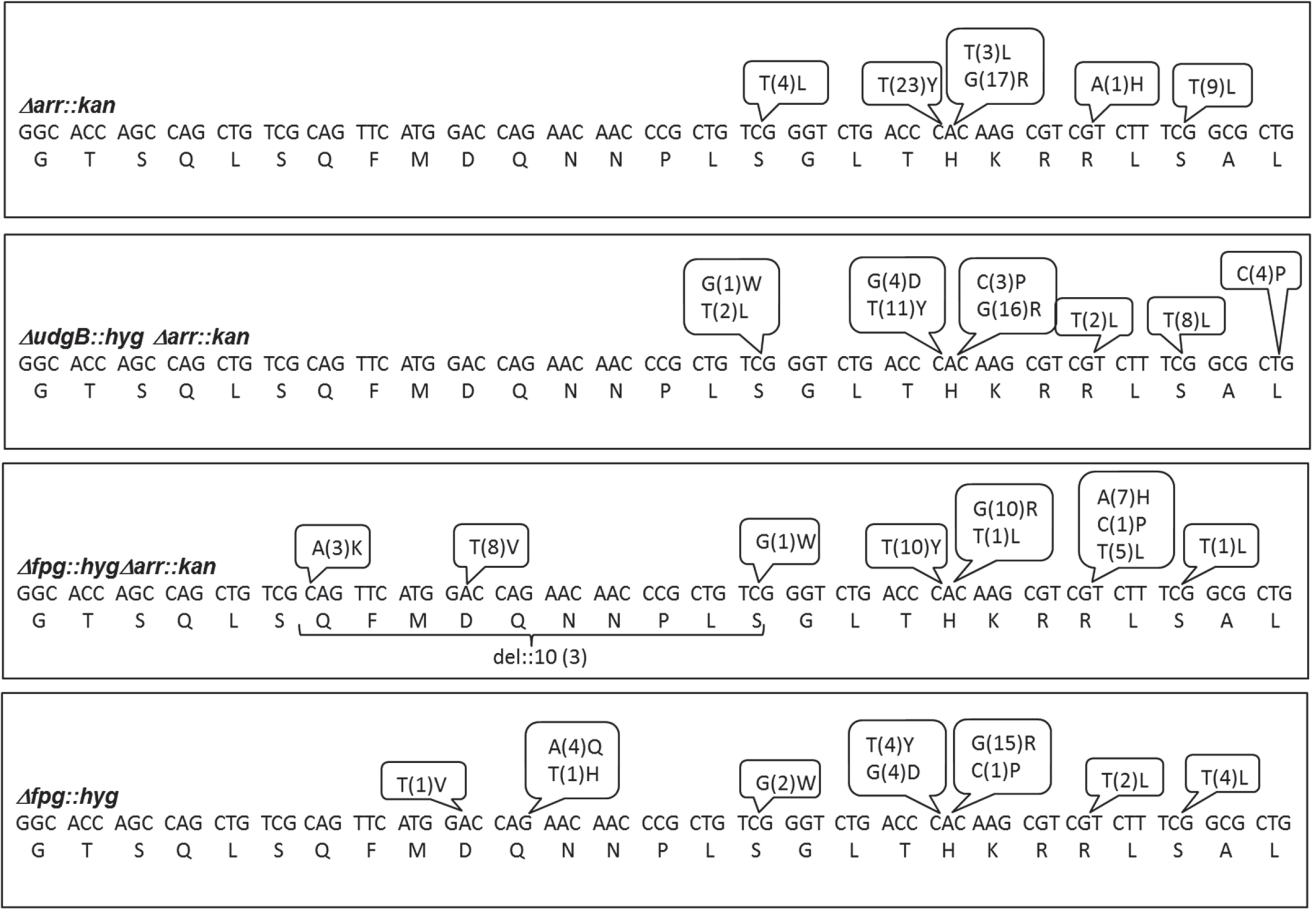
Summary of nucleotide mutations and the corresponding changes in the amino acids in RRDR loci (81 nucleotides, amino acids 426 to 452). The nucleotide mutations, corresponding amino acid changes and the frequency of occurrence of mutation in different strains are as shown.

To investigate the impact of Rif on the mutation spectrum upon introduction of the *Δarr*::*kan* allele into the *M*. *smegmatis* strains, we carried out sequence analysis of the RRDR from the spontaneous Rif^R^ isolates and compared the spectrum with that obtained from the strains wild-type for *arr*. Mutation spectrum data for *M*. *smegmatis* mc^2^155, and its *ΔudgB*::*hyg* derivatives are from the earlier reports [20, 21, 27]. As shown in Fig 7 and summarized in Table 4, mutation spectrum data for *Δarr*::*kan* revealed about 65% of C to T mutations, 30% of A to G mutations and 5% of A to T mutations, whereas those of *M*. *smegmatis* mc^2^155, wild-type for *arr* (as reported earlier) showed 52% of C to T, 34% A to G and 5% of A to T mutations. Such a mutation spectrum is very much comparable and no novel mutations or large fluctuations in the spectrum appeared in the *arr* knockout strains. Both the strains showed a predominant trend of C to T mutations, which are also the most common mutations expected for a G+C rich genome. Mutation spectrum of *ΔudgB*::*hyg* and *ΔudgB*::*hygΔarr*::*kan* is also comparable (Table 4). However, we note some changes in the mutations between the *Δfpg*::*hyg* and the *Δfpg*::*hygΔarr*::*kan* strains. For example, A to G changed from 39% to 20%, G to C from 16% to 4%, C to A from 8% to 16%, and A to T from 3% to 18%, respectively upon *arr* knockout. We may add that the mutation spectrum for *Δfpg*::*hyg* obtained in this study was found to be very similar to the one reported in our earlier study [21]. However, a major difference is that while the mutation spectrum for *Δfpg*::*hyg* was obtained at 50 μg/ml of Rif, that for *Δfpg*::*hygΔarr*::*kan* has been obtained by using a Rif concentration of merely 5 μg/ml. There is evidence to support that a mechanism that is common for the action of many antibiotics, operates through generation of reactive oxygen species [28, 29]. Thus, it is not surprising that the mutation spectrum for *Δfpg* was found to be somewhat different when lower concentration of the drug was used in the experiment (for the *Δarr*::*kan* derivative). The mutation spectrum of *M*. *smegmatis* strains particularly the *Δfpg* strains which are susceptible to oxidative stress (Fig 6) may be biased depending on the drug concentration used. In fact, it is known that at high levels of Rif, certain mutations in RRDR indeed occur with high bias [30, 31]. In addition, we noted cases of Rif resistance (6%) due to deletions in RRDR in *Δfpg*::*hygΔarr*::*kan* strain (Table 4). Such mutations for Rif^R^ were also observed by us earlier [32]. Taken together, our observations support the use of the *arr* knockout strain of *M*. *smegmatis* for Rif related phenotypes, where a low level of Rif may be used.

**Table 4:** Mutation spectrum for *M*. *smegmatis* strains.

***Mutation***	***Msm* (*wt*)**	***Δarr***	***Δfpg***	***Δfpgarr***	***ΔudgB***	***ΔudgB Δarr***
C to T or G to A	30/58 (52%)	37/57 (64.9%)	12/38 (31%)	18/50 (36%)	22/48 (45.8%)	21/51 (41.17%)
C to A or G to T	NONE	NONE	3/38 (8%)	8/50 (16%)	NONE	2/51 (3.9%)
A to G or T to C	20/58 (34%)	17/57 (29.8%)	15/38 (39%)	10/50 (20%)	22/48 (45.8%)	20/51 (39.2%)
A to C or T to G	1/58 (2%)	NONE	1/38 (3%)	NONE	3/48 (6.2%)	3/51 (5.8%)
G to C or C to G	4/58 (7%)	NONE	6/38 (16%)	2/50 (4%)	1/48 (2%)	5/51 (9.8%)
A to T or T to A	3/58 (5%)	3/57 (5.2%)	1/38 (3%)	9/50 (18%)	NONE	NONE
Del 10	NONE	NONE	NONE	3/50 (6%)	NONE	NONE
No mutation	21/79	17/74	29/67	10/60	19/67	13/64

**Note to Table 4:** Mutation spectrum data for *Msm (wt)*, *MsmΔudgB*::*hyg* (*ΔudgB*) were from our earlier studies [20, 27] and those for *MsmΔarr*::*kan* (*Δarr*), *MsmΔudgB*::*hyg Δarr*::*kan* (*ΔudgBΔarr*), *MsmΔfpg*::*hyg Δarr*::*kan* (*ΔfpgΔarr*), *MsmΔfpg*::*hyg (Δfpg*) were carried out in the present study. Numbers shown in X/Y format indicate that there were X numbers of occurrences within Y numbers of RRDR sequences. Numbers shown as % are the percent occurrences of specific mutations within the RRDR locus that showed mutations.
